# Expression of PROKR1 and PROKR2 in Human Enteric Neural Precursor Cells and Identification of Sequence Variants Suggest a Role in HSCR

**DOI:** 10.1371/journal.pone.0023475

**Published:** 2011-08-12

**Authors:** Macarena Ruiz-Ferrer, Ana Torroglosa, Rocío Núñez-Torres, Juan Carlos de Agustín, Guillermo Antiñolo, Salud Borrego

**Affiliations:** 1 Unidad de Gestión Clínica de Genética, Reproducción y Medicina Fetal, Instituto de Biomedicina de Sevilla, Hospital Universitario Virgen del Rocío/CSIC/Universidad de Sevilla, Sevilla, Spain; 2 Centro de Investigación Biomédica en Red de Enfermedades Raras (CIBERER), Sevilla, Spain; 3 Unidad de Gestión Clínica de Cirugía Infantil, Hospital Universitario Virgen del Rocío, Sevilla, Spain; Ulm University, Germany

## Abstract

**Background:**

The enteric nervous system (ENS) is entirely derived from neural crest and its normal development is regulated by specific molecular pathways. Failure in complete ENS formation results in aganglionic gut conditions such as Hirschsprung's disease (HSCR). Recently, PROKR1 expression has been demonstrated in mouse enteric neural crest derived cells and Prok-1 was shown to work coordinately with GDNF in the development of the ENS.

**Principal Findings:**

In the present report, ENS progenitors were isolated and characterized from the ganglionic gut from children diagnosed with and without HSCR, and the expression of prokineticin receptors was examined. Immunocytochemical analysis of neurosphere-forming cells demonstrated that both PROKR1 and PROKR2 were present in human enteric neural crest cells. In addition, we also performed a mutational analysis of *PROKR1*, *PROKR2*, *PROK1* and *PROK2* genes in a cohort of HSCR patients, evaluating them for the first time as susceptibility genes for the disease. Several missense variants were detected, most of them affecting highly conserved amino acid residues of the protein and located in functional domains of both receptors, which suggests a possible deleterious effect in their biological function.

**Conclusions:**

Our results suggest that not only PROKR1, but also PROKR2 might mediate a complementary signalling to the RET/GFRα1/GDNF pathway supporting proliferation/survival and differentiation of precursor cells during ENS development. These findings, together with the detection of sequence variants in *PROKR1*, *PROK1* and *PROKR2* genes associated to HSCR and, in some cases in combination with *RET* or *GDNF* mutations, provide the first evidence to consider them as susceptibility genes for HSCR.

## Introduction

The enteric nervous system (ENS) is composed of a large number of neurons and glia, which are organised into interconnected ganglia distributed throughout the gastrointestinal tract. It is originated from neural crest cells (NCCs), that invade the foregut mesenchyme during embryogenesis and migrate in a rostrocaudal direction to extensively colonize the entire length of the gut [1]. Failure in these processes results in aganglionic gut conditions, such as Hirchsprung disease (HSCR) in humans. HSCR, with an incidence of 1∶5000 live births, is the most common developmental disorder of the ENS, characterized by its incomplete formation and the absence of enteric ganglia in a variable segment of distal bowel. This leads to peristaltic misregulation and tonic contraction within the affected gut, causing intestinal obstruction [2]. HSCR most commonly presents sporadically and displays a complex pattern of inheritance with low, sex-dependent penetrance and variable expression [3].

The genetic complexity observed in HSCR could be explained by the complex nature of ENS development, which is regulated by an ever-increasing range of molecules and signalling pathways involving both the NCCs and intestinal environment [4]. Developmental biology studies have identified that the RET/GFRα1/GDNF signalling pathway is the most critical player for enteric neurogenesis and the proper expression of these proteins have been demonstrated to be crucial for the normal development of the ENS. Recently, it was shown that Prokineticin-1 (Prok1) is expressed in the mucosa and mesenchyme of the mouse embryonic gut during ENS development and promotes the survival/proliferation and differentiation, but not migration, of enteric NCCs [5]. Prok1 crosstalks with GDNF/Ret signaling and probably provides an additional layer of signaling refinement to maintain proliferation and differentiation of enteric NCCs [6].

Prokineticins (PROK1 and PROK2) belong to the AVIT protein family, a recently identified family of cysteine-rich secreted protein that share the identical amino terminal sequence crucial for their biological activities [7], [8]. These proteins are known to bind and activate two closely related Gprotein-coupled receptors, PROKR1 and PROKR2, leading to the mobilization of calcium, the stimulation of phosphoinositide-3-kinase turnover, and the activation of the mitogen activated protein kinase (MAPK) signalling pathway [9]–[11]. Prokineticins were first identified in the gastrointestinal tract as a potent agents mediating smooth muscle contraction [7]. However, they also act as survival/mitogenic factors for endothelial cells, neurons, lymphocytes and hematopoietic stem cells.

This report provides the first evidence that PROKR2 could be involved in ENS development. We show that not only PROKR1, as it was previously suggested [5], [6], but also PROKR2 are present in human enteric NCCs since both receptors are expressed in ENS progenitors isolated from ganglionic gut samples of patients diagnosed with HSCR. In addition, we have also performed a mutational analysis of *PROKR1*, *PROKR2*, *PROK1* and *PROK2* genes in a cohort of HSCR patients, evaluating them for the first time as susceptibility genes for the disease.

## Results

### Characterization of Cell Cultures

**Figure 1:**
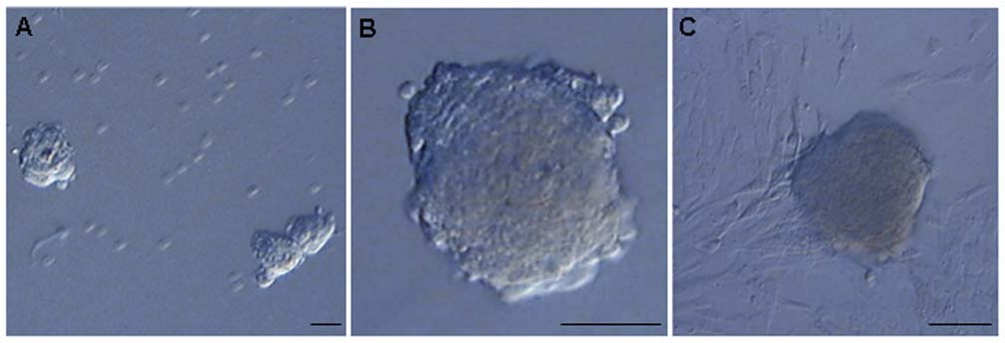
Generation of neurospheres from human neural precursors cells isolated from ENS. Phase contrast images showing characteristic neurospheres generated from freshly dissociated HSCR ganglionic gut tissue cells after 7 days in culture (A, B). Floating neurospheres were seeded onto coverslip and grown adhered using the same culture conditions (C). Scale bars 50 µm.

**Figure 2:**
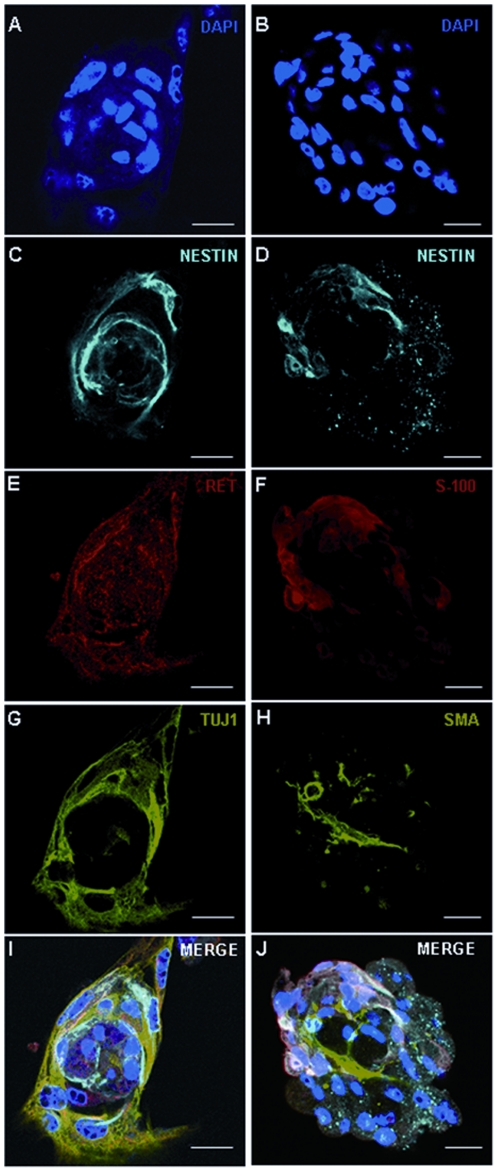
Characterization of neurospheres from human neural precursors cells isolated from ENS. Confocal microscopy images of floating neurospheres inmunostained with antibodies against Nestin (blue), TuJ1 (green), SMA (green), S100 (red) and RET (red) and counterstained with DAPI (4,6-diamidino-2-phenylindole). Scale bars 25 µm.

Postnatal ganglionic gut tissues from HSCR patients and controls were dissociated into near single cell suspension and plated in a medium suplemented with EGF, bFGF and GDNF that promotes the growth of isolated ENS progenitors. Over the next 3–4 days, floating neurospheres could be observed (Figure 1). After multiple (3–4) subcultures, part of the neurospheres derived cells still generated new neurospheres with similar characteristics, which indicates the existence of cells with self-renewal properties. To characterize the cells that form the neurospheres, they were immunostained using a neural stem cells marker (Nestin), neuronal marker (TuJ1), glial markers (GFAP, S100) and smooth muscle marker (SMA) (Figure 2). As a result, neurospheres were constituted by Nestin-positive cells (77%±2), and most of them also contained cells that expressed the neuronal marker (11%±3) (Figure S1). However, staining with antibodies against S100 and SMA showed only a small fraction of positives cells (3%±1 and 2%±0.4, respectively), while immunofluorescence for GFAP demonstrated a lack of reactivity. RET was also expressed by neurosphere cells and confocal analysis revealed the presence of this receptor in all Nestin-positive neurosphere cells. Taken together, these results suggest that the neurosphere-like bodies contained a mixture of neural and non-neural cells representing different stages of differentiation. In addition, no differences were observed between neurospheres derived from ganglionic gut from children with or without HSCR.

### Human ENS-Derived Cells Expressed PROKR1 and PROKR2 receptors “in vitro”

**Figure 3:**
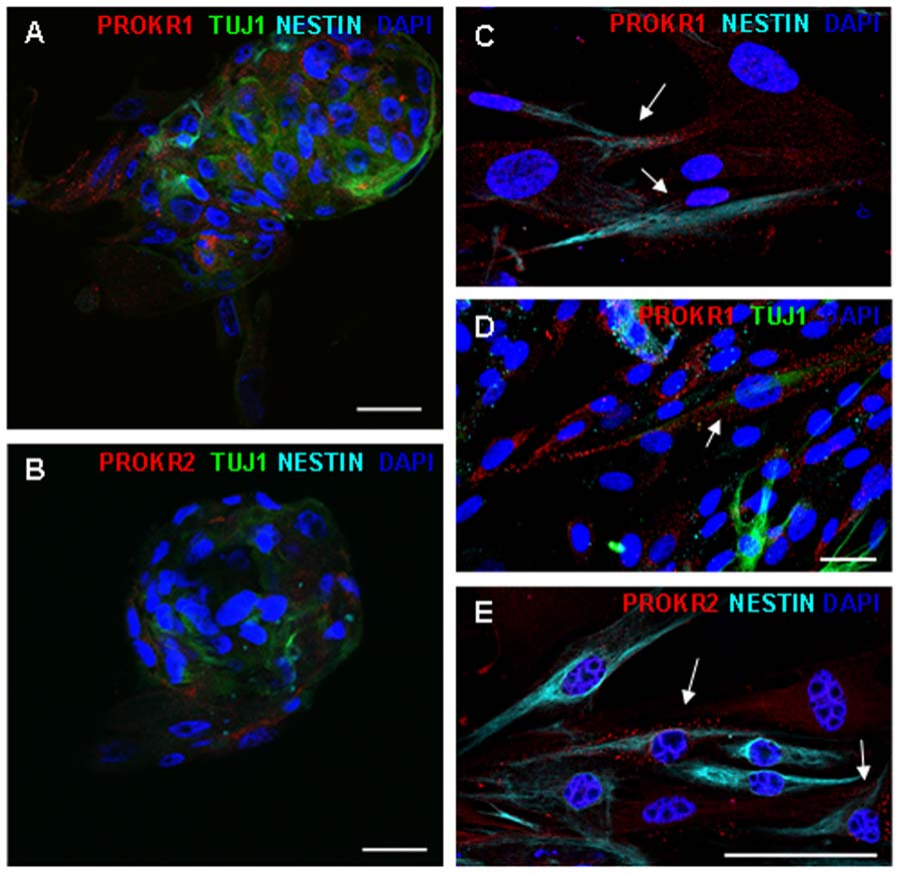
PROKR1 and PROKR2 expression in neurosphere cultures. Confocal microscopy images of floating neurospheres (A, B) and adhered neurosphere derived cells as described in Figure 1C (C–E) immunostained with antibodies against PROKR1 (red), PROKR2 (red), Nestin (blue) and TuJ1 (green). Scale bars 25 µm.

**Figure 4:**
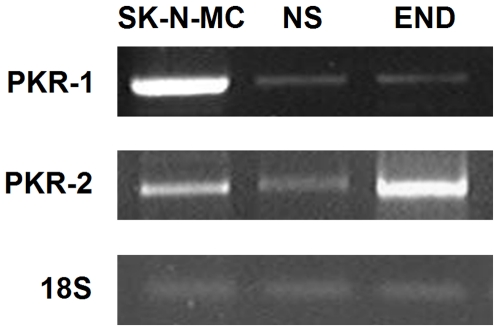
RT-PCR analysis of PROKR1 and PROKR2 expression in human ENS-derived cells. Total RNA was isolated from neurospheres (NS) and both PROKR1 and PROKR2 transcripts were detected by RT-PCR. cDNA from the neuroblastoma cell line SK-N-MC and human endometrium tissue (END) were used as positive control. Ribosomal 18S was used as the internal control.

The expression of prokineticins receptors in human neurospheres was examined. Immunocytochemical detection using specific antibodies revealed that both receptors, PROKR1 and PROKR2, were expressed in floating neurospheres obtained from the ganglionic gut from HSCR patients (Figure 3A and B, Figure S2). Cells PROKR1 and PROKR2 positives were also Nestin positive, which demonstrate that both receptors are expressed by undifferentiated enteric neural precursor cells (Figure 3C and E). In addition, we have also observed co-expression of PROKR1 with TuJ1 (Figure 3D), suggesting that this receptor is still expressed in cells already committed to neuronal fate. Analysis of mRNA by RT-PCR was also performed to confirm the presence of both PROKR1 and PROKR2 transcripts in human ENS-derived cells (Figure 4). cDNA from the neuroblastoma cell line SK-N-MC and human endometrium tissue were used as positive control and cDNA from lymphocytes as negative control (data not shown).

### Mutational analysis

**Figure 5:**
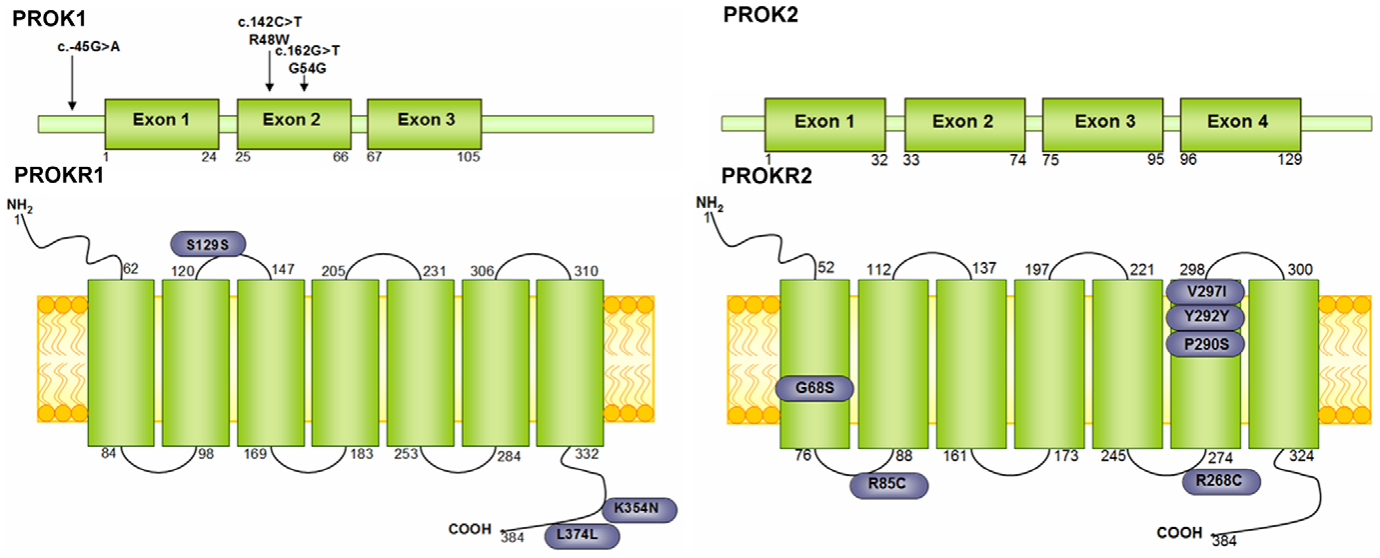
Schematic representation of PROKR1, PROK1, PROKR2 and PROK2 proteins. Distribution of the missense variants identified in our cohort of HSCR patients with respect to the predicted proteins structure using the SOSUI secondary structure prediction program.

The mutational screening of *PROKR1*, *PROK1*, *PROKR2* and *PROK2* genes revealed a total of 13 heterozygous sequence variants in 16 unrelated HSCR patients (Table 1, Figure 5). Four of those variants, detected in *PROKR2*, had been previously reported as mutations associated to Kallmann Syndrome [12]–[14]. However, no clinical signs or symptoms of Kallmann syndrome were observed in HSCR patients carrying *PROKR2* variants. Only the variants R85C in *PROKR2* and the novel G54G in *PROK1* were present in control individuals, while the remaining variants were absent in 150 control individuals tested. In addition, when genomic DNA from other family member was available, we have also analysed them and we found that all the variants had been inherited from one of their parents. Five out of the 16 patients carrying these changes (32%) also presented a mutation in the coding sequence of *RET* or *GDNF* [15], [16]. No mutations were detected in any other HSCR related gene, namely *NRTN*, *ARTN*, *PSPN*, *NTF3*, *NTRK3*, *EDNRB*, *EDN3*, *SOX10* or *PHOX2B* [16]–[21, unpublished results].

**Table 1:** *PROKR1*, *PROK1*, *PROKR2* sequence variants detected in HSCR patients.

Gene	Nucleotide Change	Amino Acid Change	Familial/Sporadic	Inheritance	Segment lenght	*In silico* analysis	Mutations in *RET* or other HSCR genes	Novel/Previously reported
*PROKR1*	c.387C>T	p.S129S	Sporadic	Not Available	S-HSCR	No effect		Novel
	c.1062A>T	p.K354N	Sporadic	Father	S-HSCR	Possibly damaging		Novel
	c.1121G>A	p.L374L	P1: SporadicP2: Familial	P1: Not AvailableP2: Mother	TCA	No effect		Novel
*PROK1*	-45G>A		Sporadic	Mother	S-HSCR	No effect	P953L in *RET* from mother	Novel
	c.142C>T	p.R48W	Sporadic	Not Available	L-HSCR	Probably damaging		rs62623571
	c.162G>T	P.G54G*	P1: SporadicP2: Sporadic	P1: MotherP2: Father	TCAS-HSCR	No effect	P1: P992L in *RET de novo*	Novel
*PROKR2*	c.202G>A	p.G68S	Sporadic	Mother	L-HSCR	Probably damaging		Novel
	c.253C>T	p.R85C*	P1: SporadicP2: Familial	P1: FatherP2: Father	S-HSCRS-HSCR	Probably damaging		Cole LW, 2008
	c.254G>A	p.R85H	Sporadic	Mother	NA	Probably damaging	V145L in *RET* from mother	Dodé C, 2006
	c.802C>T	p.R268C	Sporadic	Father	TCA	Probably damaging	G593X in *RET* from father	rs78861628
	c.868C>T	p.P290S	Sporadic	Mother	L-HSCR	Probably damaging	R93W in *GDNF* from father	Dodé C, 2006
	c.876 C>T	p.Y292Y	Sporadic	Father	S-HSCR	No effect		Novel
	c. 889G>A	p.V297I	Sporadic	Father	S-HSCR	Tolerated		Novel

S-HSCR: short segment; L-HSCR: long segment; TCA: total colonic aganglionosis; NA: not available data.

*Sequence variants detected in normal controls.

In order to establish the pathogenic relevance of those variants, we have performed *in silico* analysis using different bioinformatic tools. Based on the conserved status of the sequence and the physical properties of amino acids, Polyphen and SIFT programs predicted that, with the exception of the variant V297I in PROKR2, all the changes could generate a probably damaged protein. Moreover, DiANNA software also predicted that the inclusion of additional cysteine residues at positions 85 and 268 in PROKR2 could lead to a different distribution of the disulfide bonds in comparison to the wild type protein, that may induce a change in the three-dimensional structure of the protein. Finally, after analysis using ScanProsite interface we observed that the residues R85, R268, P290, Y292 and V297 in PROKR2 were distributed in the functional domains of the receptor, suggesting that an amino acid change at these position could affect its function.

## Discussion

Several studies have demonstrated that undifferentiated precursors cells are present within the gastrointestinal tract not only during embryonic development but also into early postnatal life [22]–[26]. In the present report, ENS progenitors were isolated from the ganglionic gut from childrens diagnosed with or without HSCR, using full-thickness gut resection specimens or gut biopsy samples, respectively. Immunocytochemical analysis of neurosphere-forming cells showed that the majority of the ENS progenitors in culture were undifferentiated neural stem cells, expressing most of them the RET receptor. A subset of neuronal cells was also observed, including TuJ1 positive cells co-expressing nestin that still retain multipotent characteristics but are competent to differentiate along the differentiated phenotype. However, only a small fraction of S100 positives cells could be identified, indicating an initial step for glial lineage differentiation in the neurospheres. In this sense, it has been demonstrated that neural diversity lineages strongly depends on the cell-intrinsic differences in their responsiveness to factors and gut NCCs are more responsive to neurogenic factors than gliogenic factors, giving rise primarily to neurons [27]. Moreover, diverse environmental conditions was suggested to play a role in regulate differentiation, and the neurosphere microenvironment in culture could be not appropriate enough for the differentiation of glial cells [24], [28]. On the other hand, it was possible to dissociate primary derived neurospheres and generate secondary and tertiary neurospheres, supporting the presence of self-renewing progenitors in culture [24].

Using these neurospheres cultures, we investigated the expression of PROKR1 and PROKR2 in human enteric NCCs. Our results show that not only PROKR1 is present in neural stem cells and neuronal precursors, but also PROKR2 receptor is observed. PROKR1 expression was previously demonstrated in mouse enteric neural crest derived cells and Prok-1 was shown to work coordinately with GDNF in the development of the ENS [5], [6]. Firstly, both GDNF and Prok-1 share common downstream elements, prominently the MAPK and Akt pathways, which provide multiple points of insertions between these two factors and lead them to exhibit similar biological functions [5]. In addition, GDNF potentiate the proliferative and differentiation effects of Prok-1 by up-regulating PROKR1 expression in enteric NCCs [6]. This functional redundancy of PROKR1/Prok-1 and RET/GFRα1/GDNF signalling supports the idea that Prok-1/PROKR1 provides a compensatory pathway to ensure the proper development of ENS.

On the other hand, our results show for the first time the expression of PROKR2 in human enteric neural crest derived cells, which confirm that expression profiles of prokineticin receptors in mouse are slightly different from that in human [29]. We suggest that PROKR2 would have a relevant role by inhibiting apoptosis of enteric neuronal precursors, as it was previously described in neural crest-derived neuroblastoma cells [30]. Therefore, PROKR2 could mediate neuronal protection or survival not only in the central nervous system [31], but also during the ENS development.

According to that, *PROKR1*, *PROK1*, *PROKR2* and *PROK2* were evaluated as susceptibility genes for HSCR, based on the etiopathogenesis of the disease. Several missense variants in *PROKR1*, *PROK1* and *PROKR2* genes were detected, most of them affecting highly conserved amino acid residues of the protein and located in functional domains of both receptors, which suggests a possible deleterious effect in their biological function. It is also worth of mentioning that four of the *PROKR2* mutations were previously described associated to Kallmann syndrome [12]–[14], another congenital disorder defined by hypogonadotropic hypogonadism and olfactory abnormalities, often associated with renal agenesis and other developmental defects. In these patients, hypogonadism is due to a failure of embryonic migration of gonadotropin-releasing hormone-synthesizing neurons from the olfactory epithelium to the forebrain, and insufficient prokineticin signalling through PROKR2 seems to play a critical role. The analysis of the functional effect of these mutations by measuring intracellular calcium release upon ligand binding has demonstrated a decreased signalling activity of the receptor [32]. Specifically, P290S impaired cell surface-targeting of the receptor and R85C, R85H and R268C presumably impaired G protein-coupling. However, when both the wild-type and mutant receptors were co-expressed, none of the mutant receptors affected the properly signalling activity provided by the wild-type receptor. Therefore, these results argue against a dominant negative effect of these mutations in vivo, supporting the current hypothesis of an autosomal recessive inheritance or oligogenic model previously reported since heterozygous mutations are not sufficient to cause Kallmann syndrome. In this sense, our results suggest that those variants detected in the genes encoding prokineticins and their receptors may also contribute to the HSCR susceptibility acting together with mutation in additional different genes, according to the additive complex model of inheritance accepted for the disease. Interestingly, we observed that mutations in *RET* proto-oncogene or *GDNF* are frequently associated to the presence of sequence variants in these genes in our cohort of HSCR patients, contributing to the manifestation of the more severed phenotypes. In summary, we show PROKR1 and PROKR2 expression in human enteric NCCs, which suggests that both prokineticin receptors might mediate a complementary signalling to the RET/GFRα1/GDNF pathway supporting proliferation/survival and differentiation of precursor cells during ENS development. These results, together with the detection of sequence variants in *PROKR1*, *PROK1* and *PROKR2* genes associated to HSCR and in some cases in combination with *RET* or *GDNF* mutation, provide the first evidence to consider them as susceptibly genes for HSCR.

## Materials and Methods

### Ethical approval

Approval from the Hospital Universitario Virgen del Rocío of Sevilla Health Ethics Subcommittee and fully written informed consent were obtained from all the participants for surgery, clinical and molecular genetic studies. The study conformed to the tenets of the declaration of Helsinki, as well as the requirements established in the Spanish law (Ley 14/2007, from 3 July 2007, “Ley de Investigación Biomedica”).

### Generation of Human Neurosphere

Human postnatal tissues of ganglionic full-thickness gut were obtained from 13 HSCR neonates (3 female, 10 male) undergoing gut resection surgery at Hospital Universitario Virgen del Rocío in Sevilla. In addition, 5 endoscopic gut biopsy samples (1 female, 4 male) from patients investigated for other gastrointestinal disorders were used as controls. From both HSCR patients and controls, age were comprised between 6 and 24 months.

All the samples were incubated in a solution of 0,26 mg/mL Trypsin Collagenase, 5 mg/mL Dispase, 0,28 mg/mL Hyaluronidase, 3,3 µg/mL Elastase and 0,6 mg/mL Collagenase in phosphate-buffered saline (PBS) for up to 30 minutes at 37°C. Digested tissue was triturated and washed, and the cells were cultured in 6-wells ultra-low attachment cluster plate. The culture medium was Dulbecco's modified Eagle medium (DMEM; 1 mg/mL Glucose) containing 100 U/mL penicillin, 100 g/mL streptomycin, supplemented with 2 mM L-glutamine (Gibco, Life Technology, California, USA), 0.05 mM 2-mercaptoethanol, 1% (v/v) N1 (Sigma Aldrich, Poole, Dorset, UK), 10% (v/v) Human serum, 20 ng/mL basic fibroblast growth factor (bFGF), 20 ng/mL epidermal growth factor (EGF) and 10 ng/mL glial cell derived neurotrophic factor (GDNF) (Peprotech, London, UK). Subcultures were performed every 7 days and experiments were performed between passage 1 and 3.

### Immunocytochemistry

For immunocytochemical studies, neurospheres were seeded onto coverslips fibronectin-poly D lysine coated and fixed with 4% (wt/vol) paraformaldehyde in 0.1 M PBS. The primary antibodies used were β-III-tubulin (TuJ1) (1∶2000; Promega Corporation, Madison), Nestin (1∶200; Santa Cruz Biotechnology, Inc.), glial fibrillary acidic protein (GFAP) (1∶1000; Dako), S100B (1∶200; Dako), α-Smooth Muscle Actin (αSMA) (1∶400; Sigma Aldrich), RET receptor (1∶250; Santa Cruz Biotechnology), Prokineticin Receptor 1 (PKR1) (1∶500) and Prokineticin Receptor 2 (PKR2) (1∶1000); (Lifespan Biosciences, Inc., Seattle, WA). The secondary antibodies used were labeled with Alexa Fluor 568 (Life Technology), Cy5 and Cy2, (Jackson Immuno Research Laboratories, Inc., West Grove, PA). The coverslips were mounted on slides with Fluoro-Gel (EMS, Hatfield, PA, USA) and fluorescent signals were detected using a Leica Spectra confocal microscope. All of the secondary antibodies were adsorbed against several species to prevent undesired cross-reactions. Omission of primary antibodies resulted in no detectable staining in all cases.

### RT-PCR

Total RNA was isolated from neurospheres and SK-N-MC cells [32] using High Pure RNA Isolation Kit (Roche Diagnostics, Mannheim, Germany), according to manufacturer instructions. 1 µg of RNA was reverse-transcribed using SuperScript™ RNA Amplification System and PCR reactions were performed using specific primers: PROKR1-F 5′-TGAGGATGTGACCAATTCCA-3′, PROKR1-R 5′-GATGGTGAAGCCGTAGAAGG-3′, PROKR2-F: 5′-CGGCAGCTCTCCTGGGAGCATGGC-3′; PROKR2-R: 5′-CGTCTGGAACCCAGGGACTGCC -3′ and 18S-F: 5′-CAGCCACCCGAGATTGAGCA-3′, 18S-R: 5′-TAGTAGCGACGGGCGGTGTG-3′. The estimated sizes of RT-PCR products were 769 for PROKR1,432 bp for PROKR2 and 253 for 18S.

### Mutational analysis

A total of 230 patients diagnosed with HSCR (23% female, 77% male) at Hospital Universitario Virgen del Rocío in Sevilla were included in the mutational analysis. 208 were sporadic cases, while 22 were familial cases belonging to 13 different families. In addition, we have also analyzed a group of 150 normal controls comprising unselected, unrelated, race, age, and sex-matched individuals.

Genomic DNA was extracted from peripheral blood leukocytes from patients and healthy controls using standard protocols. The mutational screening of the complete coding sequence and intron/exons boundaries of *PROKR1*, *PROK1*, *PROKR2* and *PROK2* was carried out by denaturing high performance liquid chromatography (dHPLC) in a WAVE DNA Fragment Analysis system (Transgenomic, Omaha, NE). In addition, those exons with aberrant profiles were subjected to sequence analysis using an ABI Prism®3730 Genetic Analyzer (Applied Biosystem, Foster City, CA) and the SeqScape® v2.5 software (Applied Biosystem, Foster City, CA).

When a novel change was detected, the appropriated DNA fragment was also screened in a group of 150 normal controls, in order to determine that such variant is not just a common polymorphism never previously described.

### Bioinformatic tools

Novel variants located within the non-coding region were submitted to several Splice Sites and Transcription Factors Binding sequences prediction interfaces such as http://www.fruitfly.org/seq_tools/splice.html; http://www.fruitfly.org/cgi-bin/seq_tools/promoter.pl; and http://www.ebi.ac.uk/asd-srv/wb.cgi. To predict the putative pathogenic role of a novel variant at the protein sequence level, we selected the SIFT, Polyphen and DiANNA tools (http://blocks.fhcrc.org/sift/SIFT.html, http://genetics.bwh.harvard.edu/pph/, http://clavius.bc.edu/~clotelab/DiANNA/). The PROKR1 and PROKR2 protein sequences were submitted to ScanProsite (http://expasy.org/tools/scanprosite/) to scan for the occurrence of patterns, profiles and motifs stored in the PROSITE database.

## Supporting Information

Figure S1 **Histogram showing distribution of Nestin+ cells, TuJ1+ cells, S-100+ cells and SMA+ cells in neurospheres.** At least 3 different preparations were assessed for each marker and 3–6 neurospheres were analysed per coveslip (∼1.300 cells). Data are presented as percentage of each phenotype with the standard error of the mean.
(TIF)

Figure S2 **Confocal microscopy images of floating neurospheres immunostained with antibodies against Nestin (blue), TuJ1 (green), PROKR1 (red), PROKR2 (red) and counterstained with DAPI (4,6-diamidino-2-phenylindole).** Scale bars 25 µm.
(TIF)
