# Supplementary figures and images for: Expression of PROKR1 and PROKR2 in Human Enteric Neural Precursor Cells and Identification of Sequence Variants Suggest a Role in HSCR
Source: PLoS One. 2011 Aug 12;6(8):e23475. doi: 10.1371/journal.pone.0023475 (PMC3155560; doi:10.1371/journal.pone.0023475)

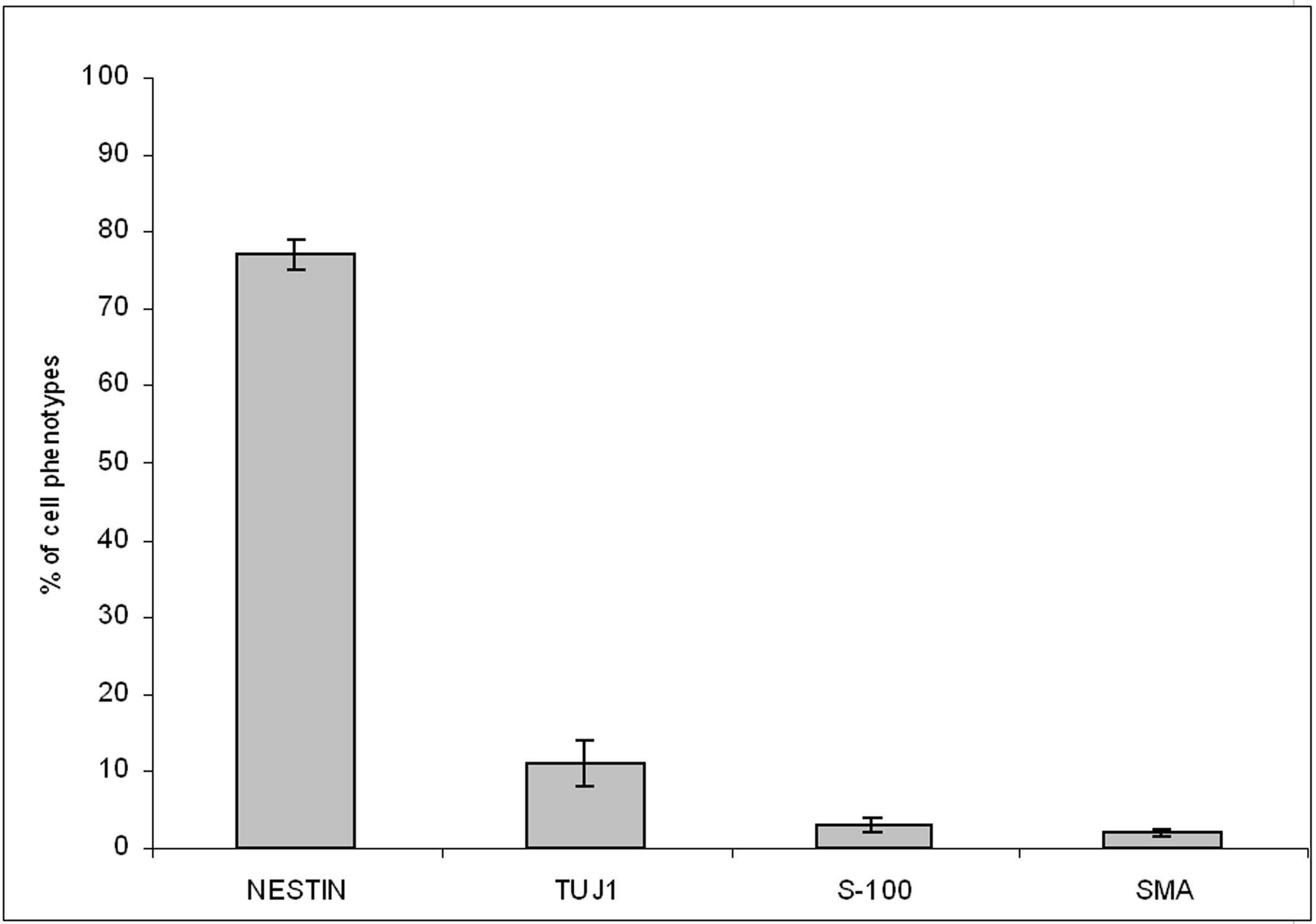

Supplement: Figure S1 — Histogram showing distribution of Nestin+ cells, TuJ1+ cells, S-100+ cells and SMA+ cells in neurospheres. At least 3 different preparations were assessed for each marker and 3–6 neurospheres were analysed per coveslip (∼1.300 cells). Data are presented as percentage of each phenotype with the standard error of the mean. (TIF) [file pone.0023475.s001.tif]

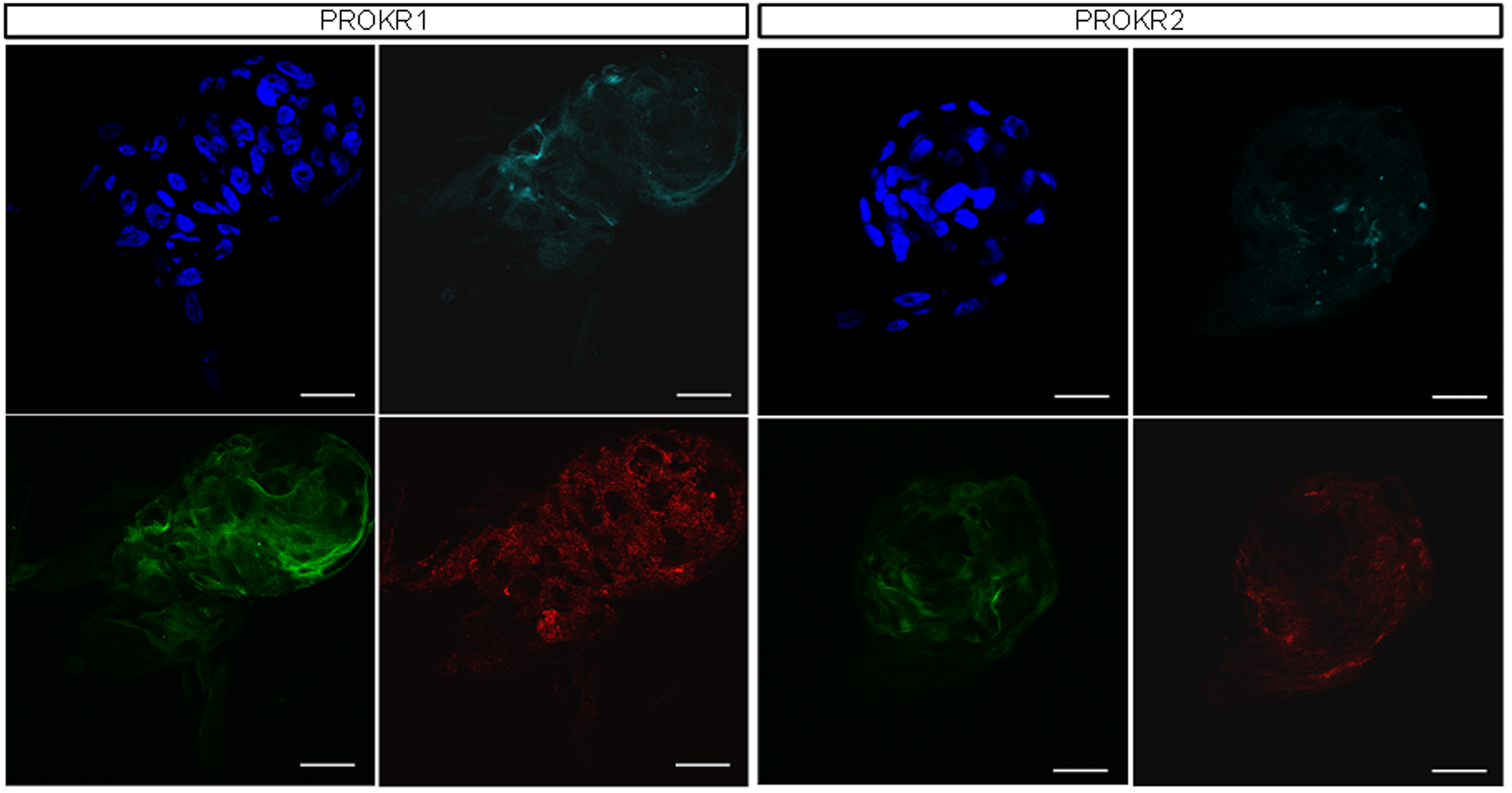

Supplement: Figure S2 — Confocal microscopy images of floating neurospheres immunostained with antibodies against Nestin (blue), TuJ1 (green), PROKR1 (red), PROKR2 (red) and counterstained with DAPI (4,6-diamidino-2-phenylindole). Scale bars 25 µm. (TIF) [file pone.0023475.s002.tif]
